# The impact of family functioning on loneliness among women during home-based postpartum confinement (Zuo Yuezi): the mediating role of social connectedness

**DOI:** 10.3389/fpsyt.2026.1761221

**Published:** 2026-06-09

**Authors:** Yi Pang, Jiejie Lin, Huijuan Lei, Fenglian Huang, Caixin Yin, Wenli Liang

**Affiliations:** 1 Department of Nursing, Guangdong Pharmaceutical University, Guangzhou, China; 2 Longgui Street Community Health Service Center, Guangzhou, China; 3 Guangzhou Medical University Guangzhou Women and Children’s Medical Center, Guangzhou, China; 4 Department of Continuing Education, Guangdong Pharmaceutical University, Guangzhou, China

**Keywords:** cultural practices, family functioning, loneliness, mediation model, postpartum period, social connectedness, Zuo Yuezi

## Abstract

**Objectives:**

This study aimed to examine the association between family functioning and loneliness among women undergoing home-based postpartum confinement in China and to assess whether social connectedness, measured by the LSNS-6, statistically accounted for this association.

**Methods:**

A cross-sectional survey was conducted among 507 postpartum women attending their 6-week follow-up visit at a maternal and child health hospital in Guangzhou, China. Participants completed the UCLA Loneliness Scale-8 (ULS-8), the Lubben Social Network Scale-6 (LSNS-6), and the Family APGAR Index. Pearson correlation analysis and mediation analysis using Hayes’ PROCESS macro (Model 4) with 5,000 bootstrap samples were performed, with stable postpartum income and parity included as covariates.

**Results:**

The prevalence of loneliness was 27.4%. Family functioning was negatively associated with loneliness (*r* = -0.514, *p* < 0.001). Higher LSNS-6 scores, indicating greater social connectedness and lower risk of social isolation, were associated with lower loneliness (*r* = -0.428, *p* < 0.001) and better family functioning (*r* = 0.419, *p* < 0.001). Mediation analysis suggested that Social Connectedness (LSNS-6) partially accounted for the association between family functioning and loneliness. Because the data were cross-sectional, this indirect pathway should be interpreted as statistical rather than causal mediation. The indirect association was significant (*B* = -0.164, 95% CI: -0.229 to -0.107), accounting for 20.50% of the total association. Women without stable postpartum income and primiparous women reported significantly higher loneliness scores.

**Conclusion:**

Loneliness was relatively common among women during home-based postpartum confinement and was associated with family functioning. Social Connectedness (LSNS-6) represented one statistical pathway linking family functioning to loneliness. Family-based support and strategies that facilitate social connectedness may be relevant to postpartum psychosocial care; however, the effectiveness of such interventions requires confirmation in longitudinal or experimental studies.

## 1 Introduction

Loneliness is a distressing subjective state that arises when individuals perceive a discrepancy between their desired and actual social relationships (1). Although loneliness, social isolation, social support, and social connectedness are closely related, they are conceptually distinct. Loneliness refers to a subjective experience, whereas social isolation reflects limited social contact or reduced network structure; social connectedness refers to the availability and accessibility of social ties that can provide emotional or practical support (2, 3). Accumulating evidence indicates that insufficient social connection is associated with adverse mental and physical health outcomes (4), and social isolation and loneliness have been linked to increased mortality risk in large-scale longitudinal evidence (5).

The postpartum period is a major life transition involving biological recovery, identity reconstruction, childcare demands, and changes in social roles. A scoping review of loneliness in pregnant and postpartum people showed that loneliness is common during the transition to parenting and is shaped by changes in identity, support, and social contact (6). The World Health Organization also emphasizes that postnatal care should address not only physical recovery but also psychological, familial, and social support needs (7). Recent longitudinal evidence further suggests that postpartum loneliness is associated with later depressive symptoms (8). In addition, insufficient social support, economic insecurity, and limited decision-making power have been identified as important risk factors for maternal loneliness and psychological distress during the postpartum period (9).

In the Chinese cultural context, postpartum social restriction has distinctive cultural and institutional features. The traditional practice of “doing the month” (Zuo Yuezi), rooted in Chinese postpartum health beliefs, establishes a family-centered model of maternal recovery (10, 11). This practice often involves remaining indoors, limiting outings, reducing household duties, and relying heavily on family members for infant care and daily support. Recent qualitative evidence also shows that Chinese mothers may continue to follow postpartum confinement practices for 4–6 weeks while seeking emotional, practical, and professional support (12). At the institutional level, China’s Special Provisions on Labor Protection of Female Employees provide a basic 98-day maternity leave, which further supports a home-based postpartum recovery structure (13). Under these combined cultural and structural conditions, postpartum women may experience a temporary contraction of external social networks. Although such restriction is culturally sanctioned and intended to promote recovery, it may also reduce opportunities for social participation and heighten vulnerability to loneliness. Maternal social isolation may also have wider family implications; for example, recent cohort evidence has linked maternal social isolation to behavioral problems in preschool children (14).

Based on this background, the following hypothesis was proposed:

H1: Greater social connectedness is associated with lower levels of loneliness. Conceptually, this corresponds to the expectation that a lower risk of social isolation is associated with lower loneliness.

When external social interactions are restricted, the family becomes the primary source of emotional and practical support. Bowlby’s attachment theory proposes that close relationships can provide a secure base and a safe haven in times of distress (15). In the perinatal period, attachment-related vulnerability may be particularly salient because women experience physical recovery, role transition, and increased dependence on close relationships. Previous evidence suggests that anxious aspects of insecure attachment, especially preoccupation with relationships, are associated with depressive symptoms during pregnancy and the postpartum period (16). During Zuo Yuezi, when external contact is reduced and physical recovery requires assistance, the quality of family relationships may therefore be especially important for postpartum emotional well-being. According to the stress-buffering model, social support can reduce the negative psychological impact of stressful circumstances (17). In this context, supportive family relationships may buffer distress associated with physical recovery, role transition, and restricted social contact by providing emotional validation, practical help, and reassurance.

Family functioning refers to the overall capacity of family members to communicate, coordinate roles, provide emotional support, and solve problems collaboratively (18). Perinatal evidence suggests that perceived social support is closely related to maternal mental health (19), and family-oriented approaches have been explored as feasible supports for postpartum psychological difficulties (20). Earlier postpartum research also indicates that family and marital functioning are important aspects of maternal adjustment (21). Evidence from other populations also suggests that family functioning is closely related to loneliness and broader psychosocial adjustment (22). In addition, family networks may play an important protective role against social isolation, with recent evidence among the oldest-old showing that family ties differ from friendship ties in their relationship with isolation risk (23). Recent qualitative research on postpartum care in China also emphasizes the importance of interpersonal connections and support in maternal well-being (24).

In the present study, these perspectives are integrated into a family-embedded social connectedness framework. During Zuo Yuezi, postpartum women’s daily interactions are reorganized around the household, and family functioning may shape both intrafamilial emotional security and access to broader social ties. Bowlby’s attachment theory and the stress-buffering model help explain the direct intrafamilial pathway: responsive family interactions, emotional validation, shared caregiving responsibility, and practical reassurance may reduce mothers’ distress and meet their needs for closeness, security, and recognition.

Social connectedness is proposed as the mediator because Zuo Yuezi specifically restricts women’s access to social contacts beyond the immediate household. Unlike marital quality, partner responsiveness, or emotional validation, which mainly reflect intrafamilial processes, social connectedness captures the availability and accessibility of broader family and friend networks. In a well-functioning family, family members may facilitate mothers’ communication with friends, peers, community healthcare providers, or online maternal support groups while still respecting postpartum recovery practices. Conversely, poor family functioning may narrow mothers’ accessible social networks and make the discrepancy between desired and available relationships more salient.

Self-discrepancy theory provides a psychological explanation for why inadequate support may be associated with loneliness: emotional distress may arise when individuals perceive a gap between their actual experiences and their expected or ideal self-state (25). This logic is consistent with the cognitive discrepancy model of loneliness, which views loneliness as arising partly from a perceived gap between desired and actual social relationships (1). During Zuo Yuezi, a mother may expect family closeness, understanding, and practical help, while also experiencing reduced external contact and increased childcare demands. When family functioning is poor and social connectedness is limited, this discrepancy may become more salient, thereby intensifying loneliness. Conversely, well-functioning families may reduce this discrepancy by providing emotional validation, responsive communication, and opportunities for maintaining appropriate social contact.

Therefore, partial mediation was theoretically expected rather than only observed *post hoc*. Family functioning may be associated with loneliness directly through emotional security, recognition, and family responsiveness, and indirectly through mothers’ social connectedness. This integrated framework is particularly relevant to Zuo Yuezi because the family becomes not only a source of care but also a gatekeeper of mothers’ access to wider social relationships. Therefore, the present study examines whether social connectedness statistically accounts for the association between family functioning and loneliness among women during home-based postpartum confinement in China.

Accordingly, the following hypotheses were further proposed:

H2: Higher levels of family functioning are associated with lower levels of loneliness.

H3: Higher levels of family functioning are associated with greater social connectedness.

H4: Social connectedness partially accounts for the association between family functioning and loneliness in this cross-sectional statistical mediation model, reflecting an indirect social-network pathway in addition to direct intrafamilial emotional pathways.

## 2 Materials and methods

### 2.1 Study design and participants

This cross-sectional study used convenience sampling. From August 2022 to December 2023, postpartum women were recruited from the 42-day postpartum outpatient clinic of a tertiary maternal and child health hospital in Guangzhou, China. Women who met the eligibility criteria and agreed to participate received a paper-based questionnaire after providing written informed consent. A total of 517 questionnaires were distributed. Four participants withdrew before completing the questionnaire, and six questionnaires were excluded because of patterned or identical responses. Finally, 507 valid questionnaires were included in the analysis, yielding an effective response rate of 98.1% among distributed questionnaires.

Sample size estimation was based on Kendall’s empirical rule, which recommends that the sample size should be 10–20 times the number of variables included in the analysis (26). Considering the number of variables assessed and allowing for approximately 20% invalid responses, the estimated sample size ranged from 276 to 552. The final sample size of 507 was within this range. However, this rule-of-thumb approach does not replace an *a priori* power analysis based on expected indirect effects; this issue is acknowledged as a methodological limitation.

#### 2.1.1 Inclusion criteria

Participants were eligible if they met the following criteria: (1) within 6 weeks postpartum; (2) aged ≥ 20 years and married; (3) clear consciousness and ability to independently complete the survey; and (4) provided written informed consent and agreed to participate voluntarily.

#### 2.1.2 Exclusion criteria

Participants were excluded if they: (1) had recently experienced major stressful life events, such as severe illness or death of a close relative; (2) had a history of diagnosed psychological disorders; or (3) experienced mother–infant separation after childbirth.

#### 2.1.3 Elimination criteria

Questionnaires with patterned responses or identical answers across all items were excluded from analysis.

### 2.2 Measures

#### 2.2.1 General information questionnaire

A self-developed questionnaire was used to collect basic information based on the study aims. It included: (1) sociodemographic characteristics, including age, per capita monthly household income, stable postpartum income, medical insurance status, family structure, and enrollment in family doctor services; and (2) obstetric characteristics, including primiparous or multiparous status, gestational age, pregnancy complications, history of adverse pregnancy outcomes, history of chronic disease, infant feeding method, employment of a postpartum caregiver (Yuesao), and receipt of postpartum home visits.

#### 2.2.2 UCLA loneliness scale–short form

Loneliness was assessed using the 8-item short form of the UCLA Loneliness Scale (ULS-8), developed by Hays and DiMatteo (27) and later translated and validated in Chinese by Zhou et al. (28). Items are rated on a 4-point Likert scale. Positively worded items are scored from 1 (“Never”) to 4 (“Often”), whereas reverse-worded items are scored inversely. Total scores range from 8 to 32, with higher scores indicating greater loneliness. A score >16 was used to estimate the presence of loneliness. This cut-off was used as a screening threshold rather than a clinical diagnostic criterion; to our knowledge, it has not been specifically validated among Chinese postpartum women. Therefore, the prevalence estimate should be interpreted as a screening-based estimate. In the present study, Cronbach’s alpha was 0.803.

#### 2.2.3 Lubben social network scale–6

Social connectedness was measured using the 6-item Lubben Social Network Scale (LSNS-6), developed by Lubben et al. (29) and validated in Chinese by Chang et al. (30). The LSNS-6 includes two subscales assessing family networks and friend networks, with three items in each subscale. Each item is rated on a 6-point scale from 0 (“None”) to 5 (“Nine or more”), yielding a total score ranging from 0 to 30. Higher scores indicate larger and more supportive social networks, that is, greater social connectedness and lower risk of social isolation; scores below 12 indicate risk of social isolation. To avoid conceptual confusion, the raw LSNS-6 score was analyzed and reported as Social Connectedness (LSNS-6) throughout the Results, tables, and figure. In the present study, Cronbach’*s* alpha was 0.820.

#### 2.2.4 Family APGAR index

Family functioning was measured using the Family APGAR Index, developed by Smilkstein et al. (31) and introduced for Chinese clinical use by Lü and Gu (32). The scale contains five items assessing Adaptation, Partnership, Growth, Affection, and Resolve. Each item is rated on a 3-point Likert scale from 0 (“Hardly ever”) to 2 (“Almost always”). Total scores range from 0 to 10, with higher scores indicating better perceived family functioning. Scores of 0–3 indicate severe family dysfunction, 4–6 indicate moderate family dysfunction, and 7–10 indicate good family functioning. In the present study, Cronbach’s alpha was 0.887.

### 2.3 Procedure

Researchers provided standardized instructions explaining the study purpose, significance, voluntary nature of participation, and questionnaire completion requirements. Paper-based questionnaires were distributed onsite, completed independently by participants, and collected immediately afterward. Researchers reviewed questionnaires onsite for completeness. When item-level omissions were identified before questionnaire submission, participants were invited to complete the omitted items voluntarily. Questionnaires with patterned or identical responses were excluded. No statistical imputation was performed, and no item-level missing data remained in the final analytic dataset. The entire survey process required approximately 10–15 minutes.

### 2.4 Statistical analysis

All data were analyzed using SPSS version 26.0. Continuous variables were expressed as mean ± standard deviation ( $\bar{x}$ ± *s*), and categorical variables were expressed as frequency and percentage. Differences in loneliness scores across participant characteristics were examined using independent-samples *t*-tests or one-way analysis of variance, as appropriate. Pearson correlation analysis was performed to examine associations among family functioning, Social Connectedness (LSNS-6), and loneliness.

Mediation analysis was conducted using the SPSS PROCESS macro version 4.1 (Model 4), with 5,000 bootstrap resamples to generate 95% confidence intervals (CI). Stable postpartum income and parity were included as covariates because they were significantly associated with loneliness in univariate analyses. Indirect associations were considered statistically significant when the bootstrap 95% CI did not contain zero. The significance level was set at α = 0.05. Given the cross-sectional design, the analysis was interpreted as statistical mediation rather than evidence of temporal or causal mediation (33).

Common method variance was assessed using Harman’s single-factor test (34). All items from the ULS-8, LSNS-6, and Family APGAR Index were entered into an unrotated exploratory factor analysis. The first unrotated factor accounted for 34.05% of the total variance, below the commonly used 40% threshold. This result did not suggest a dominant single-factor structure; however, it cannot completely rule out potential inflation caused by shared self-report measurement.

## 3 Results

### 3.1 General characteristics of participants

A total of 507 postpartum women completing home-based recovery were included in the final analysis. The mean age of participants was 31.18 ± 3.91 years. Most participants reported a per capita monthly household income of 5,000–9,999 RMB (67.7%), 59.0% lived in stem families, and 70.4% were not enrolled in family doctor services.

Loneliness scores ranged from 8 to 25, with a mean score of 14.14 ± 3.86. Among all participants, 139 (27.4%) met the criterion for loneliness (score > 16). Univariate analyses showed that women without stable postpartum income had significantly higher loneliness scores than those with stable income (*t* = -2.949, *p* = 0.003). In addition, primiparous women reported significantly higher loneliness scores compared with multiparous women (*t* = 2.489, *p* = 0.013). No other demographic or obstetric variables demonstrated significant differences in loneliness scores (**Table 1**).

**Table 1:** General characteristics and univariate analysis of loneliness scores among postpartum women (n = 507).

Variable	Overall	Loneliness score ( $\bar{x}$ ± *s*)	*t*/*F* value	*P* value
Age			-0.094	0.925
20∼34	410(80.9)	14.13 ± 3.90		
≥35	97(19.1)	14.18 ± 3.70		
Per capita monthly household income (CNY)			0.647	0.524
<5000	108(21.3)	13.86 ± 3.81		
5000∼9999	343(67.7)	14.28 ± 3.83		
≥10000	56(11.0)	13.86 ± 4.16		
Stable postpartum income			-2.949	**0.003**
Yes	417(82.2)	13.91 ± 3.79		
No	90(17.8)	15.22 ± 4.02		
Medical insurance coverage			-1.360	0.175
Yes	479(94.5)	14.20 ± 3.86		
No	28(5.5)	13.18 ± 3.84		
Family structure			-0.223	0.824
Nuclear family	208(41.0)	14.10 ± 3.88		
Stem family	299(59.0)	14.17 ± 3.85		
Enrollment in family doctor service			-1.522	0.129
Yes	150(29.6)	13.74 ± 3.56		
No	357(70.4)	14.31 ± 3.98		
Primipara			2.489	**0.013**
Yes	311(61.3)	14.48 ± 3.99		
No	196(38.7)	13.61 ± 3.59		
Gestational age			0.474	0.635
28∼36	30(5.9)	14.47 ± 4.21		
≥37	477(94.1)	14.12 ± 3.84		
Presence of pregnancy complications			0.671	0.503
Yes	164(32.3)	13.98 ± 3.95		
No	343(67.7)	14.22 ± 3.82		
History of adverse pregnancy outcomes			-0.594	0.553
Yes	135(26.6)	14.31 ± 3.87		
No	372(73.4)	14.08 ± 3.86		
History of chronic disease(*s*)			-0.636	0.525
Yes	35(6.9)	14.54 ± 3.71		
No	472(93.1)	14.11 ± 3.87		
Infant feeding method			0.919	0.399
Breastfeeding	212(41.8)	13.91 ± 3.69		
Mixed feeding	262(51.7)	14.37 ± 4.00		
Formula feeding	33(6.5)	13.85 ± 3.87		
Employment of a postpartum caregiver (Yuesao)			-1.094	0.275
Yes	131(25.8)	13.82 ± 4.15		
No	376(74.2)	14.25 ± 3.75		
Received postpartum home visit(*s*)			-1.253	0.211
Yes	441(87.0)	14.06 ± 3.80		
No	66(13.0)	14.70 ± 4.26		

Data are presented as *n* (%) or mean ± SD. Differences in loneliness scores were examined using independent-samples *t*-tests or one-way analysis of variance, as appropriate. Bold values indicate statistically significant differences (P < 0.05).

### 3.2 Correlations among family functioning, social connectedness, and loneliness

Correlation analysis showed that family functioning (7.75 ± 2.43) was negatively correlated with loneliness (*r* = -0.514, *p* < 0.001). Higher Social Connectedness (LSNS-6) scores were associated with lower loneliness (*r* = -0.428, *p* < 0.001) and better family functioning (*r* = 0.419, *p* < 0.001). Because higher LSNS-6 scores indicate greater social connectedness and lower risk of social isolation, the negative association between Social Connectedness (LSNS-6) and loneliness is consistent with H1. In other words, women with more limited social networks reported higher loneliness scores. Detailed correlation coefficients are shown in **Table 2**.

**Table 2:** Correlations among family functioning, social connectedness, and loneliness (r values).

Item	1	2	3
Family functioning	—		
Social Connectedness	0.419***	—	
Loneliness	-0.514***	-0.428***	—

***p < 0.001. Higher LSNS-6 scores indicate greater social connectedness and lower risk of social isolation.

### 3.3 Testing the mediating role of social connectedness between family functioning and loneliness

#### 3.3.1 Mediation analysis

After controlling for stable postpartum income and parity, family functioning was negatively associated with loneliness (*B* = -0.800, *SE* = 0.061, *p* < 0.001). Family functioning was positively associated with Social Connectedness (LSNS-6) (*B* = 0.774, *SE* = 0.076, *p* < 0.001). When Social Connectedness (LSNS-6) was added to the model, it was negatively associated with loneliness (*B* = -0.211, *SE* = 0.034, *p* < 0.001), and the association between family functioning and loneliness decreased but remained significant (*B* = -0.636, *SE* = 0.064, *p* < 0.001). These findings suggest that Social Connectedness (LSNS-6) partially accounted for the association between family functioning and loneliness after adjustment for stable postpartum income and parity. Regression model details are presented in **Table 3**.

**Table 3:** Regression analysis of factors associated with loneliness and social connectedness among postpartum women (n = 507).

Variable	Model for total effect on loneliness			Model for social connectedness			Model for loneliness (with mediator)		
	*B*	*SE*	*t*	*B*	*SE*	*t*	*B*	*SE*	*t*
Stable postpartum income	0.897	0.384	2.337*	-0.723	0.484	-1.494	0.744	0.371	2.005*
Parity	-0.743	0.300	-2.475*	0.340	0.379	0.898	-0.671	0.290	-2.316*
Family Functioning	-0.800	0.061	-13.216***	0.774	0.076	10.146***	-0.636	0.064	-9.927***
Social Connectedness	—	—	—	—	—	—	-0.211	0.034	-6.198***
*R²*	0.280			0.180			0.331		
*F*	65.304***			36.814***			62.227***		

*B* = unstandardized regression coefficient; *SE* = standard error; *p < 0.05, ***p < 0.001.

Stable postpartum income was coded as 1 = yes and 2 = no.

Parity was coded as 1 = primiparous and 2 = multiparous.

All models were adjusted for stable postpartum income and parity.

The models estimate associations rather than causal effects.

#### 3.3.2 Mediating role of social connectedness

**Figure 1:**
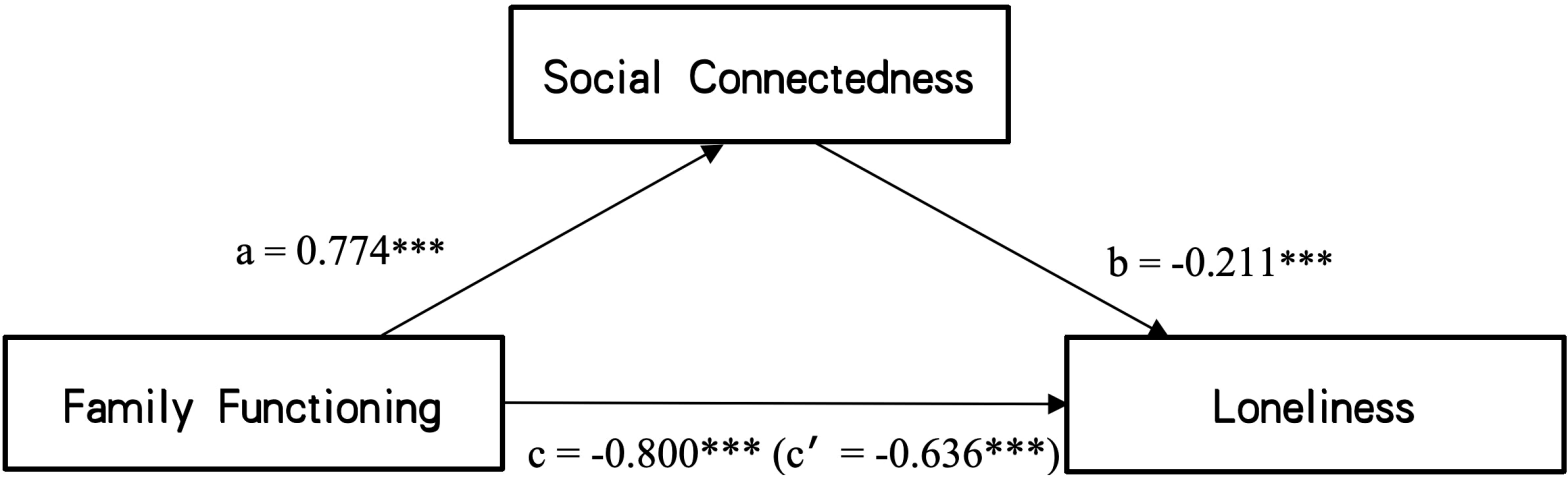
Statistical mediation model of social connectedness in the association between family functioning and loneliness. Note: Coefficients are unstandardized regression coefficients adjusted for stable postpartum income and parity. *a* = path from family functioning to social connectedness; *b* = path from social connectedness to loneliness; *c* = total association; c′ = direct association after including social connectedness. ***p < 0.001.

As shown in **Table 4**, the direct association between family functioning and loneliness was -0.636 (95% CI: -0.762 to -0.510). The indirect association through Social Connectedness (LSNS-6) was -0.164 (95% CI: -0.229 to -0.107), accounting for 20.50% of the total association. These results indicate modest partial statistical mediation: Social Connectedness (LSNS-6) represented one pathway through which family functioning was associated with loneliness, while most of the association remained direct or may operate through other unmeasured mechanisms. The statistical mediation model is illustrated in **Figure 1**.

**Table 4:** Decomposition of total, direct, and indirect associations through social connectedness.

Path	Unstandardized effect (*B*)	Boot *SE*	95% CI		Proportion of total effect
			Lower	Upper	
Indirect effect	-0.164	0.031	-0.229	-0.107	20.50%
Direct effect	-0.636	0.064	-0.762	-0.510	79.50%
Total effect	-0.800	0.061	-0.919	-0.681	100%

The indirect association was estimated using 5,000 bootstrap samples. Because of the cross-sectional design, this table presents statistical indirect associations rather than causal mediation effects.

## 4 Discussion

### 4.1 Status and characteristics of loneliness among postpartum women at home

In this study, 27.4% of women undergoing home-based postpartum confinement reported loneliness. This finding is broadly consistent with existing evidence showing that loneliness is common during pregnancy, early parenthood, and the postpartum transition (6). The transition to motherhood involves physical recovery, sleep disruption, role adaptation, and a temporary restructuring of daily social life. Therefore, loneliness during the postpartum period may reflect not only the number of available social contacts but also mothers’ perceived mismatch between expected and actual support.

Within the Chinese sociocultural context, this experience may be shaped by Zuo Yuezi. Although postpartum confinement is intended to promote recovery and family care, it often requires women to remain at home, reduce outings, and limit face-to-face contact with wider social networks. Such practices may temporarily shift women from active social roles to primarily domestic and caregiving roles. When the available family and social support does not meet mothers’ emotional or practical needs, this restriction may intensify loneliness.

Because this study did not evaluate intervention effectiveness, practical implications should be interpreted cautiously. Nevertheless, the findings suggest that alternative pathways for social connection may be worth considering in postpartum care. Community healthcare services and medical institutions may consider culturally sensitive digital platforms, hybrid support options, or peer-support approaches to provide professionally facilitated psychosocial support for postpartum women (35). Such approaches may enable mothers to share experiences, receive professional guidance, and maintain appropriate social connection while reducing barriers to in-person contact during confinement.

Women without stable postpartum income reported higher loneliness scores. This finding is consistent with recent evidence that economic insecurity is an important social determinant of perinatal mental health (36). Lack of stable income may reduce financial autonomy, increase dependence on family members, and heighten concerns about childcare and household expenses, thereby increasing psychological burden and loneliness. Primiparous women also reported higher loneliness scores than multiparous women. First-time mothers may face greater uncertainty, lower parenting confidence, and stronger disruption of pre-birth social roles. Prior qualitative studies have described first-time motherhood as a period marked by reduced social contact, identity disruption, emotional fragility, and uncertainty (37–39). Therefore, women without stable income and primiparous women may require more targeted emotional, informational, and family-based support during postpartum confinement.

### 4.2 Correlations among loneliness, family functioning, and social connectedness

This study found that better family functioning was associated with lower loneliness. During postpartum confinement, mothers’ daily care and emotional support are largely embedded within the family environment. Families with better functioning may provide more responsive communication, clearer role sharing, and more reliable emotional and practical assistance. These family resources may help meet mothers’ needs for belongingness, security, and recognition, thereby reducing loneliness. This interpretation is consistent with evidence showing that family ties play an important role in social isolation (22) and with perinatal research suggesting that family functioning is closely linked to maternal psychosocial well-being (40).

Family functioning was also positively associated with social connectedness. This finding suggests that well-functioning families may not only provide internal support but also help mothers maintain appropriate connections with external networks. During Zuo Yuezi, mothers’ opportunities to communicate with friends, access professional guidance, join peer groups, or use community resources may depend partly on family support and household arrangements. Integrating family functioning assessment and family guidance into postpartum services may therefore be relevant for improving the social context of postpartum recovery (41).

Higher social connectedness was associated with lower loneliness. Because this construct reflects larger and more supportive social networks, mothers with stronger social connectedness experienced less loneliness, whereas women with more limited networks reported higher loneliness scores. During postpartum confinement, limited access to meaningful social ties may increase the discrepancy between mothers’ desired and available social relationships, especially when emotional validation and peer understanding are insufficient. Thus, social connectedness may represent an important proximal social-network context for postpartum loneliness.

### 4.3 The partial mediating role of social connectedness in the association between family functioning and loneliness

The mediation analysis showed that social connectedness statistically accounted for 20.50% of the association between family functioning and loneliness. This finding supports the theoretically expected partial, rather than complete, mediation model. In the context of Zuo Yuezi, family functioning may be associated with loneliness through at least two complementary pathways. The first is an intrafamilial emotional pathway, in which responsive communication, shared caregiving responsibility, partner or family recognition, and emotional validation may directly satisfy mothers’ needs for closeness and security. The second is a broader social-network pathway, in which well-functioning families may help mothers maintain accessible and supportive ties with friends, peers, healthcare professionals, and online or community maternal support resources.

Social connectedness was therefore specified as the mediator because it captures this second pathway more directly than other family-related constructs. Marital quality, partner responsiveness, emotional validation, and parenting self-efficacy are important but conceptually different mechanisms: they mainly describe the quality of internal family interactions or maternal psychological resources. By contrast, social connectedness reflects the size and perceived availability of family and friend networks, which is especially relevant when postpartum confinement temporarily restricts women’s external social participation. The modest proportion mediated suggests that social connectedness is a meaningful but not exhaustive pathway. Most of the association may still operate through unmeasured intrafamilial emotional, cognitive, or practical mechanisms.

The indirect pathway is theoretically plausible in the context of Zuo Yuezi. Well-functioning families may encourage mothers to maintain contact with friends, peers, healthcare professionals, or online maternal communities while still respecting postpartum recovery practices. These opportunities may enhance mothers’ perceived social connectedness and reduce loneliness. At the same time, family functioning may also be directly associated with loneliness because emotional communication, empathy, and shared responsibility within the family can satisfy mothers’ immediate needs for closeness and support.

However, this mediation result should be interpreted as a statistical indirect association rather than evidence of a causal process. The cross-sectional design does not establish whether family functioning preceded social connectedness or loneliness, nor can it exclude reverse or reciprocal relationships. For example, mothers experiencing loneliness may perceive family functioning more negatively or withdraw from social networks. Longitudinal studies are needed to clarify temporal ordering and test whether changes in family functioning predict later changes in social connectedness and loneliness.

### 4.4 Limitations

This study has several limitations. First, the cross-sectional design precludes definitive causal interpretation. Although the mediation model was theoretically driven, the temporal ordering of family functioning, social connectedness, and loneliness cannot be established. The mediation findings therefore reflect statistical decomposition rather than causal mediation. Longitudinal or experimental designs are needed to clarify directionality.

Second, participants were recruited from a single tertiary maternal and child health hospital in Guangzhou and were primarily urban postpartum women. The findings may not generalize to rural populations, other Chinese regions, ethnic minority groups, or women with different socioeconomic and family structures. In addition, the total number of potentially eligible women attending the clinic during the recruitment period was not recorded, which limits assessment of selection bias. Future studies should consider multicenter sampling and more detailed participant-flow reporting.

Third, all key variables were collected from the same respondents using self-report questionnaires at a single time point, which may introduce common method variance, recall bias, and social desirability bias. Although Harman’s single-factor test did not indicate a dominant single-factor structure, this test is only a preliminary diagnostic procedure and is not sufficient to rule out common method bias. Therefore, the observed associations may still have been inflated by shared measurement methods. Future research should use stronger procedural and statistical remedies, such as temporal separation of measurements, multi-informant assessments, spouse-reported family functioning, clinician-rated psychosocial indicators, or objective indicators of social interaction where feasible.

Fourth, the study focused on the first 42 days postpartum, a culturally specific period of physical recovery and confinement. The results therefore provide a snapshot of early postpartum experiences but do not capture changes across the broader postpartum year. Longitudinal tracking from late pregnancy through the first year postpartum would provide a more comprehensive understanding of how loneliness and social connectedness evolve over time.

Fifth, the ULS-8 cut-off score of >16 was used to estimate loneliness prevalence. To our knowledge, this threshold has not been specifically validated among Chinese postpartum women. Therefore, the prevalence of 27.4% should be interpreted as a screening-based estimate rather than a clinically validated diagnostic classification.

Sixth, the mediation model adjusted only for stable postpartum income and parity because these variables were significantly associated with loneliness in the univariate analyses. However, several highly relevant factors were not measured or adjusted for, including depressive symptoms, anxiety, sleep quality, marital relationship quality or marital satisfaction, partner responsiveness, emotional validation, childcare burden, parenting self-efficacy, and online social support. These omitted variables may produce residual confounding and may lead to overestimation or underestimation of the observed associations among family functioning, social connectedness, and loneliness. This is particularly important because postpartum depressive and anxiety symptoms are closely related to loneliness, perceived family functioning, and social connectedness. Therefore, the internal validity of the mediation model should be interpreted cautiously. Future studies should incorporate a broader set of theory-driven covariates and test alternative or competing mediation pathways.

Seventh, the sample size was estimated using Kendall’s empirical rule rather than an *a priori* power analysis based on expected indirect effects. Although the final sample size was adequate for the planned analyses according to this rule-of-thumb, future studies should conduct power analyses or simulation-based sample size estimation for mediation models.

This report was revised with reference to STROBE guidance for cross-sectional observational studies (42).

## 5 Conclusions

This study found that loneliness was relatively common among women during home-based postpartum confinement in China. Better family functioning was associated with lower loneliness, and this association was partially accounted for by higher Social Connectedness (LSNS-6). These findings suggest that both intrafamilial emotional processes and broader social-network contexts are relevant to postpartum loneliness during Zuo Yuezi.

The results should be interpreted cautiously because the study used a cross-sectional design and cannot establish causal or intervention effects. Nevertheless, the findings highlight the potential importance of assessing family functioning, identifying women with limited social connectedness, and considering culturally appropriate strategies to support postpartum social connection. Integrating family-based assessment with community or digital support resources may be a promising direction for postpartum psychosocial care, but its effectiveness should be tested in future longitudinal and intervention studies.

## Data Availability

The raw data supporting the conclusions of this article will be made available by the authors, without undue reservation.
